# Atomic-Scale Mechanisms of Nanoscale Material Removal in FeCrNiCoCu High-Entropy Alloys: Coupled Effects of Crystallography, Grain Size, and Composition

**DOI:** 10.3390/nano16110675

**Published:** 2026-05-28

**Authors:** Xu Ling, Peng Fu, Yan Li, Zhiqiang Zhou, Zhuo Li

**Affiliations:** 1 College of Intelligent Control Engineering, Hunan Chemical Vocational Technology College, Zhuzhou 412000, China; 2 College of Mechanical and Vehicle Engineering, Hunan University, Changsha 410082, China; 3 Beijing Institute of Mechanical Equipment, Beijing 100000, China; 4 College of Civil Engineering, Hunan University, Changsha 410082, China

**Keywords:** FeCrNiCoCu high-entropy alloys, molecular dynamics simulation, dislocation behavior, crystallographic orientation, elemental composition

## Abstract

High-entropy alloys, due to their excellent mechanical properties and service stability, hold broad application prospects under extreme working conditions. However, their high strength and complex multi-component characteristics also pose significant processing challenges. This study investigates the nanoscale material removal mechanisms of single-crystal and polycrystalline FeCrNiCoCu high-entropy alloys (HEAs) under abrasive scratching using molecular dynamics simulations. In single-crystal HEAs, dislocations preferentially nucleate along <110> directions, with significant lattice self-healing and elastic recovery. Crystallographic orientation strongly affects dislocation density, phase transformation, and residual plastic deformation, with the (100) plane exhibiting the most favorable machining performance. For polycrystalline HEAs, subsurface deformation is dominated by dislocation migration, grain boundary rupture, and dislocation entanglement, leading to higher dislocation density, larger residual plastic deformation, and increased phase transformation compared with single crystals. Elemental composition significantly modulates these behaviors: higher Cu and Cr contents suppress dislocation motion and reduce subsurface defects, improving surface quality, whereas higher Fe content slightly increases plastic deformation but mitigates phase transformation and amorphization. Grain size effects are also pronounced, with smaller grains showing higher dislocation density and residual deformation. These findings provide atomic-scale insights into the combined effects of crystallography, grain size, and elemental composition on the machining response of FeCrNiCoCu HEAs, offering guidance for precision machining and alloy design.

## 1. Introduction

High-entropy alloys (HEAs) are a novel class of multi-principal-element materials that exhibit excellent mechanical strength, wear resistance, thermal stability, and corrosion resistance owing to their severe lattice distortion, sluggish diffusion effect, and complex atomic interactions [1,2]. Among various HEA systems, FeCrNiCoCu alloys have attracted considerable attention because they combine a relatively stable face-centered cubic (FCC) structure with strong compositional tunability and excellent strength–ductility balance [3]. In particular, the coexistence of multiple transition-metal elements in FeCrNiCoCu alloys leads to pronounced local lattice distortion and heterogeneous deformation behavior, making this alloy system highly sensitive to crystallographic orientation, grain structure, and elemental composition during mechanical deformation and material removal processes. Compared with conventional FCC metals such as Cu and Ni, FeCrNiCoCu HEAs exhibit more complex dislocation evolution behavior, enhanced lattice stability, and stronger resistance to plastic deformation and subsurface damage under severe loading conditions. These characteristics make FeCrNiCoCu alloys promising candidate materials for high-end engineering applications requiring excellent wear resistance and surface integrity, such as aerospace components, precision molds, and extreme-environment mechanical systems [4,5,6].

However, the high strength and hardness of HEAs also lead to considerable challenges in machining processes, such as large cutting forces and severe tool wear [7]. In addition, the low thermal conductivity and complex phase structures of HEAs tend to cause rapid temperature rise in the cutting zone, pronounced work hardening, and difficulty in controlling subsurface damage [8]. Furthermore, multiphase microstructures and elemental segregation may result in unstable machining processes and increased variability in machining quality [9]. Therefore, a systematic investigation of the machining mechanisms and damage evolution behaviors of HEAs is of great scientific significance for improving their machining quality and efficiency, and for promoting their engineering applications.

At present, ultra-precision machining technologies for HEAs mainly include high-efficiency cutting using cubic boron nitride (CBN) superhard tools, precision grinding with high-performance grinding wheels, and various non-traditional machining methods [10]. Liu et al. employed polycrystalline cubic boron nitride (PCBN) tools to cut WNbMoTaZr HEAs. Although improved surface quality was achieved through process optimization, the material removal rate remained low, and residual stresses and crack damage were observed in the subsurface [11]. Zhang et al. [12] investigated the tool wear mechanisms during machining of WNbMoTaZr_0.5_ refractory HEAs using YT15 cemented carbide tools, and found that the multi-component elemental composition of HEAs leads to adhesive and diffusion wear of the cutting tool, which is further aggravated by excessive cutting speed and temperature. Liang et al. [13] studied the influence of Al content on tool wear during micro-milling of FeCoNiCrAl HEAs, revealing that a moderate reduction in Al content can effectively suppress adhesive wear, oxidative wear, and edge chipping, thereby improving machining quality. Xing et al. [14] applied laser-assisted diamond cutting to FeCoNiCrMn HEAs and characterized the subsurface microstructural evolution using advanced techniques. Their results indicated that the laser–friction coupled thermal effect enhances atomic ordering within grains and reduces subsurface damage. Nevertheless, although laser-based non-traditional machining techniques offer high precision and flexibility, they are often limited by large heat-affected zones and low machining efficiency.

Molecular dynamics (MD) simulation is a numerical method based on interatomic potential functions, capable of directly describing lattice evolution and dynamic behaviors of materials at the atomic scale. It provides an effective approach for investigating deformation and material removal mechanisms in complex multi-component HEA systems. Previous studies have explored dislocation evolution, phase transformation, and subsurface damage formation during nanocutting and nanoscratching processes in Cu, Ni, Al alloys, and HEA systems [15,16,17,18]. Nguyen et al. [19] systematically studied the effects of temperature, strain rate, and grain size on the deformation mechanisms of AlCoCrCuFeNi HEAs using MD simulations, revealing the underlying dislocation activities during plastic deformation. Wu et al. [20] developed an MD model of single-abrasive scratching on FeCrNiCoCu HEAs to analyze the effects of scratching speed, depth, and temperature on material removal mechanisms. Wang et al. [21] explored the influence of surface micro-topography on the scratching-induced deformation behavior of CoCrFeMnNi HEAs, demonstrating that fine groove structures on the surface can effectively reduce plastic deformation damage and friction coefficient. Although existing studies have preliminarily revealed the lattice evolution mechanisms and nanoscale material removal behaviors of HEAs during precision machining from an atomic perspective, the effects of multi-component composition, crystallographic orientation, and grain characteristics on their machining mechanisms remain unclear and require further in-depth investigation.

Despite considerable progress in experimental and simulation studies on HEA machining, the atomic-scale mechanisms underlying deformation, dislocation evolution, and material removal in multi-component alloys remain incompletely understood. In particular, the effects of crystallographic orientation, grain size, and multi-element composition on machining behavior have yet to be fully elucidated. In this study, molecular dynamics simulations are employed to systematically investigate the scratching-induced lattice evolution, dislocation activity, phase transformation, and atomic displacement in single-crystal and polycrystalline FeCrNiCoCu high-entropy alloys. The findings aim to provide fundamental insights into the machining mechanisms of HEAs at the atomic scale, offering guidance for optimizing processing strategies and enhancing the surface integrity and performance of these advanced alloys.

It should be noted that MD simulations inherently operate at nanometer length scales and picosecond timescales, with deformation rates far exceeding those in practical machining processes. As a result, they cannot fully capture long-range stress propagation, large-scale thermal transport, or collective microstructural evolution in real engineering systems. In addition, the finite simulation domain, simplified boundary conditions, and approximate interfacial interaction models may influence dislocation motion and defect interactions, limiting the direct representation of full thermomechanical and tribochemical complexity. Nevertheless, MD simulations remain a powerful tool for revealing atomic-scale deformation mechanisms and for establishing qualitative relationships between microstructural characteristics and nanoscale material response in high-entropy alloys.

## 2. Simulation Methodology

Molecular dynamics (MD) simulations of CBN abrasive scratching on FeCrNiCoCu high-entropy alloys were performed using the Large-scale Atomic/Molecular Massively Parallel Simulator (LAMMPS) (LAMMPS 64-bit 29Aug2024-MSMPI, USA) [22]. The simulation results were subsequently analyzed and visualized using the OVITO software (OVITO 3.15.4).

### 2.1. Material Model Construction of FeCrNiCoCu High-Entropy Alloy

#### 2.1.1. Single-Crystal FeCrNiCoCu High-Entropy Alloy Model

**Figure 1:**
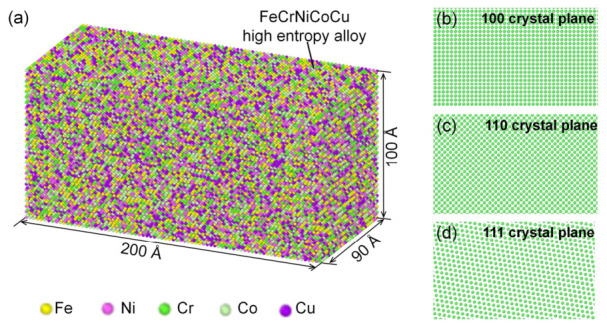
Atomic model of the single-crystal FeCrNiCoCu high-entropy alloy and atomic arrangements on different crystallographic planes: (**a**) overall material model; (**b**) atomic arrangement on the (100) plane; (**c**) atomic arrangement on the (110) plane; (**d**) atomic arrangement on the (111) plane.

The FeCrNiCoCu high-entropy alloy is a multi-principal-element system composed of Fe, Ni, Cr, Co, and Cu in equiatomic or near-equiatomic proportions, typically exhibiting a face-centered cubic (fcc) crystal structure [23]. In this study, the material model of the FeCrNiCoCu alloy was constructed using the Atomsk software (12 January 2024) [24], as illustrated in Figure 1a. The model dimensions are 200 × 90 × 100 Å (length × width × height), containing approximately 138,750 atoms. The model size was selected to minimize boundary effects while maintaining computational feasibility. The present study did not systematically investigate the influence of system size on the simulation results because of the high computational cost associated with large-scale nanoscratching simulations of multi-component high-entropy alloys. The adopted model dimensions were selected according to previously reported molecular dynamics studies on metallic nanoscratching and nanomachining processes, where similar model scales were widely used to capture defect evolution and subsurface deformation behavior. All atoms in the model are arranged in an fcc lattice, with Fe, Ni, Cr, Co, and Cu atoms distributed in an equiatomic ratio of 1:1:1:1:1. Considering that Cu has the largest lattice constant among the constituent elements (3.615 Å) [25], the lattice constant of the constructed HEA model was set to 3.615 Å.

In the present study, the FeCrNiCoCu high-entropy alloy was constructed as an idealized random solid-solution model using the Atomsk software. Fe, Cr, Ni, Co, and Cu atoms were randomly assigned to FCC lattice sites according to the prescribed atomic fractions, thereby generating statistically random local atomic environments within the alloy. The present model does not employ the Special Quasirandom Structure (SQS) approach [26]. Instead, a fully random substitution strategy was adopted to efficiently represent the lattice distortion and compositional disorder characteristics of the high-entropy alloy at large simulation scales. Such random solid-solution models have been widely used in large-scale molecular dynamics simulations of HEAs because they can effectively capture the stochastic local chemical environments and deformation heterogeneity while maintaining computational feasibility during dynamic deformation and machining simulations. In contrast, SQS methods are primarily intended for reproducing near-random correlation functions in first-principles and equilibrium thermodynamic calculations. Since the primary objective of the present work is to investigate relative defect evolution and nanoscratching-induced deformation mechanisms rather than equilibrium thermodynamic properties, the adopted random solid-solution approximation is considered physically reasonable for the current MD simulations. It should nevertheless be noted that local chemical short-range order and elemental clustering, which are neglected in the present model, may influence stacking-fault energy, dislocation mobility, and defect evolution in real HEAs. These effects will be considered in future investigations using more sophisticated chemically ordered models.

To investigate the influence of compositional variations on the machining mechanisms, additional models with varying elemental contents of Fe, Cr, and Cu were established. For the baseline equiatomic FeCrNiCoCu alloy, each element has an atomic fraction of 20%. When the fraction of a selected element (e.g., Fe) is reduced to 10%, the proportions of the remaining elements are adjusted accordingly to maintain the overall composition balance, as shown in Table 1. The same approach was applied to construct models with varying Cr and Cu contents. Due to the pronounced anisotropy of single-crystal FeCrNiCoCu HEAs, crystallographic orientation plays a critical role in their material removal behavior. Therefore, three different crystallographic orientations, namely (100), (110), and (111), were constructed, as shown in Figure 1b–d, to systematically investigate the effect of crystal orientation on the machining mechanisms of the alloy.

#### 2.1.2. Polycrystalline FeCrNiCoCu High-Entropy Alloy Model

**Figure 2:**
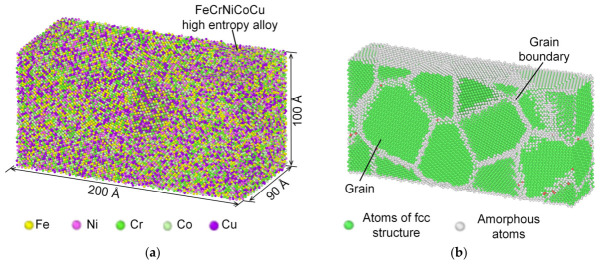
Polycrystalline FeCrNiCoCu high-entropy alloy model: (**a**) overall material model; (**b**) grain morphology within the polycrystalline structure.

A polycrystalline FeCrNiCoCu high-entropy alloy model was constructed using the Atomsk software, as shown in Figure 2a. In this model, Fe, Ni, Cr, Co, and Cu atoms are distributed in an equiatomic ratio of 1:1:1:1:1, and the overall dimensions are 200 × 90 × 100 Å (length × width × height). The polycrystalline models contained approximately 152,462 atoms depending on the grain size and grain boundary configuration. As illustrated in Figure 2b, the grain orientations within the polycrystalline model are randomly distributed, while atoms inside each grain are arranged in a regular face-centered cubic (fcc) lattice. To investigate the effect of grain size on the scratching mechanisms of the alloy, three polycrystalline models with different average grain sizes were constructed, with grain sizes of 35 Å, 40 Å, and 45 Å, respectively. The selected grain sizes represent typical nanocrystalline structures within the computationally accessible range of molecular dynamics simulations. These nanoscale grains enable sufficient grain boundary interactions and defect evolution to be captured during scratching while maintaining reasonable computational efficiency. Previous studies have shown that nanometer-scale grain sizes strongly influence dislocation motion, grain boundary deformation, and subsurface damage behavior. Therefore, grain sizes of 35 Å, 40 Å, and 45 Å were selected to investigate the grain-size-dependent deformation mechanisms of FeCrNiCoCu high-entropy alloys during nanoscratching.

### 2.2. Scratching Model

**Figure 3:**
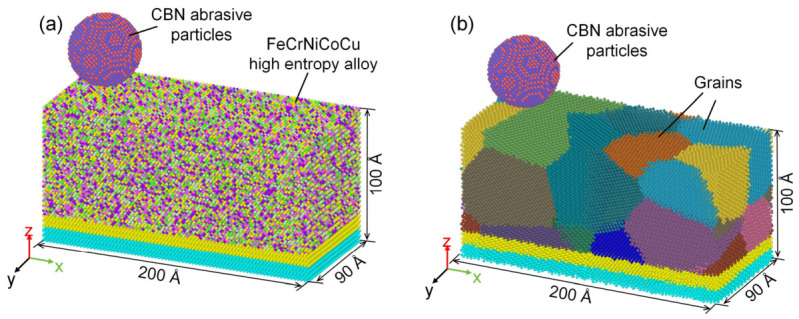
Scratching models: (**a**) MD simulation model of single-abrasive scratching on single-crystal FeCrNiCoCu high-entropy alloy; (**b**) MD simulation model of single-abrasive scratching on polycrystalline FeCrNiCoCu high-entropy alloy.

An MD simulation model of abrasive scratching on single-crystal FeCrNiCoCu high-entropy alloy was established using the LAMMPS software, as shown in Figure 3a. The abrasive particle is made of cubic boron nitride (CBN), which has a cubic crystal structure with a lattice constant of 3.615 Å. The abrasive is modeled as a rigid spherical particle with a radius of 25 Å.

The abrasive particle is initially positioned at the middle position along the y-direction. Along the z-direction, the distance between the bottom of the abrasive and the top surface of the alloy is set to 5 Å. To ensure the stability of the simulation system, both the workpiece and the abrasive are divided into three regions: a fixed layer, a thermostat layer, and a Newtonian layer. The fixed layer is used to constrain the workpiece and to drive the motion of the abrasive during scratching. The temperature of the constant temperature layer is controlled by a Nose Hoover thermostat [27], with an initial temperature of 297 K (room temperature) and a damping parameter of 0.1 ps. Room temperature was selected because it is commonly used in experimental machining processes and molecular dynamics studies of nanoscratching behavior in metallic materials and high-entropy alloys. In addition, maintaining a constant room-temperature condition helps isolate the effects of crystallographic orientation, grain size, and elemental composition on the deformation behavior during scratching simulations. Atoms in the Newtonian layer follow Newton’s laws of motion and undergo lattice transformation and atomic migration under external loading. Considering the nanoscale nature and finite dimensions of MD simulations, periodic boundary conditions were applied along the x and y directions to reduce artificial free-surface effects and improve numerical stability, while a non-periodic boundary condition was imposed along the z direction to allow surface deformation during scratching. Nevertheless, due to the limited simulation domain, these boundary conditions may still influence stress distribution, dislocation propagation, and defect interactions within the subsurface region. Therefore, the present simulations mainly aim to reveal relative deformation trends and atomistic mechanisms rather than quantitatively reproducing macroscopic machining responses. Figure 3b shows the simulation model of CBN abrasive scratching on polycrystalline FeCrNiCoCu HEA. Except for the use of a polycrystalline workpiece, all other simulation settings are identical to those in Figure 3a.

To eliminate the influence of initial internal stresses within the material and ensure the accuracy of the simulation results, the system was first relaxed for 10,000 timesteps under the NVE ensemble with a timestep of 1 fs prior to scratching. The penetration depth of the abrasive into the alloy was set to 0.75 nm, the scratching velocity was 2 Å/ps, and the total scratching distance was 120 Å. The scratching velocity used in the present MD simulations is significantly higher than that employed in practical machining processes. Such elevated deformation rates are commonly adopted in molecular dynamics studies due to the intrinsic limitation of accessible simulation timescales. Simulating experimentally realistic scratching velocities would require prohibitively large computational resources and simulation times. It should be noted that high scratching velocities may induce localized temperature rise, stress concentration, and transient density fluctuations near the abrasive–workpiece interface. Therefore, the absolute values of friction force, defect density, and subsurface damage obtained in this study should not be directly interpreted as quantitative predictions for macroscopic machining systems. Instead, the present simulations mainly aim to reveal the relative trends and atomistic deformation mechanisms associated with crystallographic orientation, grain size, and elemental composition in FeCrNiCoCu high-entropy alloys. The detailed parameters used in the MD simulations are summarized in Table 2.

### 2.3. Interatomic Potential Functions

Interatomic potentials are mathematical models used to describe the interactions between atoms and their lattice behavior in MD simulations. Commonly used potential types include the embedded atom method (EAM), extended Tersoff-type potentials (ExTeP), and Lennard–Jones (LJ) potentials [28]. The interatomic potentials employed in this study are described as follows.

The interactions among Fe, Ni, Cr, Co, and Cu atoms in the FeCrNiCoCu high-entropy alloy are modeled using the ZBL-modified EAM potential developed by Deluigi et al. [29]. This potential effectively overcomes the inaccuracy of conventional EAM potentials at short interatomic distances by incorporating the Ziegler–Biersack–Littmark (ZBL) universal repulsive potential. It has been demonstrated to accurately capture lattice phase transformations and dislocation evolution during the deformation and machining processes of FeCrNiCoCu HEAs. The mathematical expressions of this potential are given in Equations (1) and (2).

(1) $$V_{ZBL}\left(r\right)=\frac{Z_{1}Z_{2}e^{2}}{4\pi \varepsilon_{0}r}\cdot \varphi \left(\frac{r}{a}\right)$$

(2) $$U=\sum F_{i}(\rho_{i})+\frac{1}{2}\sum f_{ij}(r_{ij})$$

where V represents the total energy of ZBL potential energy, Z1 and Z2 are the atomic numbers of two atoms, r is the interatomic distance, e is the charge of an electron, ε0 is the vacuum dielectric constant, ϕ(x) is the shielding function, a is the shielding length, U represents the total energy of EAM potential energy, Fi represents the embedding energy, the second term represents the pair potential, ρi is the sum of the densities of the electron clouds generated at the i-th atom by the extra-nuclear electrons of all other atoms, fij is the function of the pair potential action between the i-th and j-th atoms, and rij is the distance between the i-th and j-th atoms.

The ExTeP potential function developed by Los et al. [30] is used to describe the interaction between B and N atoms, which can stably represent the lattice morphology of cubic boron nitride. The main parameters of this potential function are as follows (Table 3).

Among them, Eb is the binding energy, B2D is the bulk modulus, μ2D is the two-dimensional shear modulus, E2D is the Young’s modulus, μ2D is the Poisson’s ratio, and C11,2D is the elastic constant.

The L-J potential energy function is used to describe the interactions between five metal atoms (including Fe, Cr, Ni, Co, Cu) and B/N atoms. For the L-J potential function, the formula for calculating the atomic potential energy of the entire system is as follows [31]:

(3) $$V(r_{ij})=4\varepsilon \left[{\left(\frac{\sigma }{r_{ij}}\right)}^{12}+{\left(\frac{\sigma }{r_{ij}}\right)}^{6}\right]$$

In Equation (3), rij represents the distance between atoms i and j. ε is the potential well, indicating the strength of the interaction potential. σ represents the interatomic distance when the interaction potential value is zero. The specific parameters of the L-J potential are listed in Table 4, with a cutoff distance of 12 Å [32,33].

It should be emphasized that the Lennard–Jones (LJ) potential used for the metal–CBN cross-interactions provides a simplified description of the abrasive–workpiece interface, primarily capturing short-range, non-bonded interactions between the abrasive and the alloy surface. Although this approach has been widely adopted in molecular dynamics nanoscratching simulations of metallic systems due to its computational efficiency and numerical stability, it does not explicitly account for more complex interfacial phenomena such as adhesion, charge transfer, bond formation and rupture, oxidation, or tribochemical reactions that may occur in realistic scratching processes. Accordingly, the present simulations are intended to qualitatively describe the relative deformation behavior and defect evolution mechanisms of FeCrNiCoCu high-entropy alloys, rather than to quantitatively reproduce the exact tribochemical response at the abrasive–workpiece interface.

## 3. Results and Discussion

**Figure 4:**
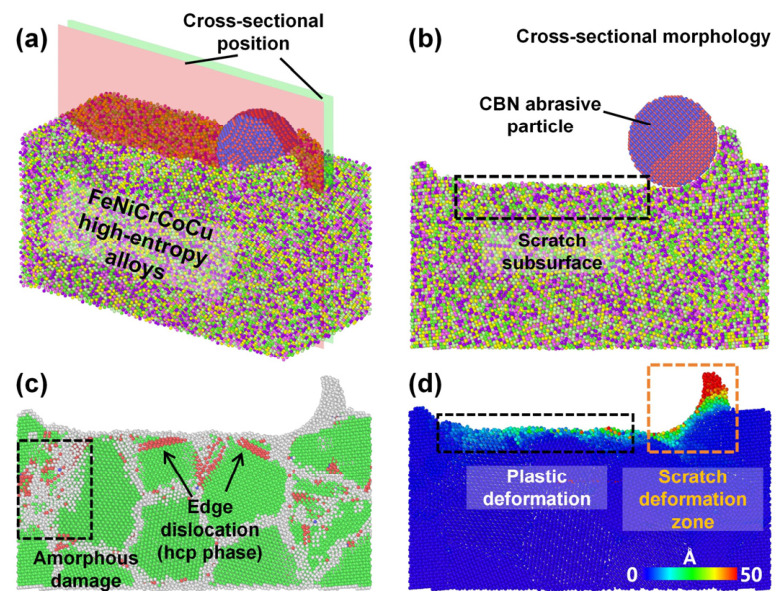
Cross-sectional location and morphology, lattice structure, and atomic displacement of the scratched subsurface. (**a**) Cross-sectional location; (**b**) subsurface morphology; (**c**) lattice structure of the cross-section; (**d**) atomic displacement contour of the cross-section. (Polycrystalline model, grain size: 40 Å).

Figure 4a shows the three-dimensional morphology of the scratched surface of polycrystalline FeCrNiCoCu high-entropy alloys after CBN abrasive scratching, where a distinct groove is formed on the alloy surface. To analyze the material removal mechanism, it is necessary to examine the subsurface lattice evolution and atomic displacement behavior. Therefore, a representative observation cross-section, as shown in Figure 4b, was extracted from the scratched model using OVITO to investigate the lattice characteristics of the subsurface region. The location of the cross-section is indicated in Figure 4a. It is parallel to the scratching direction and positioned at the middle position of the model along the y-axis, with a thickness of 10 Å. Common neighbor analysis (CNA) and atomic displacement analysis were performed on this cross-section using OVITO [34], and the results are presented in Figure 4c,d. The results indicate that the subsurface region of the polycrystalline alloy exhibits pronounced amorphization and dislocation defects after scratching, with dislocation atoms predominantly existing in the hcp phase. In addition, significant plastic deformation occurs in both the subsurface region and the scratching deformation zone. Therefore, analyzing subsurface lattice morphology, phase transformation, and atomic displacement is an effective approach to elucidate the material removal mechanism of FeCrNiCoCu high-entropy alloys. In this study, all subsequent cross-sectional analyses are conducted at the same location as that shown in Figure 4a.

### 3.1. Single-Crystal FeCrNiCoCu High-Entropy Alloy

#### 3.1.1. Material Removal Mechanism of Single-Crystal FeCrNiCoCu High-Entropy Alloy

**Figure 5:**
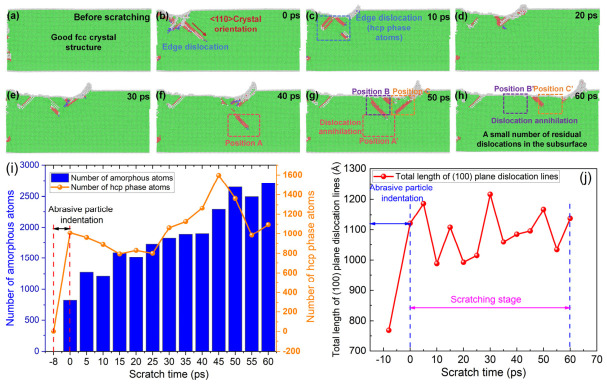
Evolution of subsurface lattice structure and phase-transformed atoms during scratching on the (100) surface. Subsurface lattice configurations at different scratching times: (**a**) before scratching; (**b**) 0 ps; (**c**) 10 ps; (**d**) 20 ps; (**e**) 30 ps; (**f**) 40 ps; (**g**) 50 ps; (**h**) 60 ps. (**i**) Variation in the number of amorphous and hcp atoms induced by the abrasive as a function of scratching time. (**j**) Evolution of the total dislocation line length in the subsurface during scratching.

The lattice evolution and atomic displacement behavior during the scratching process of the FeCrNiCoCu high-entropy alloy were analyzed using the common neighbor analysis (CNA) and atomic displacement methods implemented in OVITO. The results are shown in Figure 5. As illustrated in Figure 5a, prior to scratching, the (100) surface of the HEA exhibits a perfect face-centered cubic (fcc) lattice structure, with no obvious subsurface lattice defects observed. As shown in Figure 5b, with the indentation of the CBN abrasive, pronounced edge dislocations begin to nucleate along the <110> crystallographic direction in the subsurface region. In fcc materials, dislocations are typically identified by the formation of local hexagonal close-packed (hcp) structures. Figure 5c,d demonstrate that, as the scratching time increases to 20 ps, the dislocations migrate along with the movement of the scratching-induced deformation zone. As shown in Figure 5e–h, dislocations at positions A, B, and C in the subsurface region undergo annihilation as the deformation zone progresses during scratching. This phenomenon indicates that the subsurface lattice of the FeCrNiCoCu HEA possesses a strong defect annihilation and structural recovery. Upon removal of the external load, the distorted lattice structures containing dislocations gradually recover toward a stable fcc configuration. As shown in Figure 5h, the subsurface region largely retains a well-ordered fcc structure after scratching, with only a small number of residual dislocations. Furthermore, Figure 5i shows that the number of amorphous atoms and hcp-phase atoms induced by abrasive scratching increases progressively during both the indentation and scratching processes. Since dislocations in fcc materials are closely associated with hcp atomic structures, the number of hcp atoms can be used as an indicator of dislocation density in the subsurface. It is also evident from Figure 5i that the increase in hcp atoms mainly occurs during the indentation stage, while no significant growth is observed during the scratching stage. Consistently, Figure 5j shows that the total length of dislocation lines follows a similar trend, with no obvious increase during scratching.

Overall, the MD simulation results indicate that, on the (100) surface of the FeCrNiCoCu HEA, dislocations preferentially nucleate along the <110> direction under external loading. Once the external force is removed, the induced dislocations tend to annihilate. During scratching, dislocations are primarily localized within the deformation zone beneath the abrasive, with no significant dislocation multiplication observed. After scratching, only a small number of residual dislocations and phase transformations remain in the subsurface. These findings confirm that the FeCrNiCoCu high-entropy alloy exhibits excellent toughness and strong lattice structural recovery.

**Figure 6:**
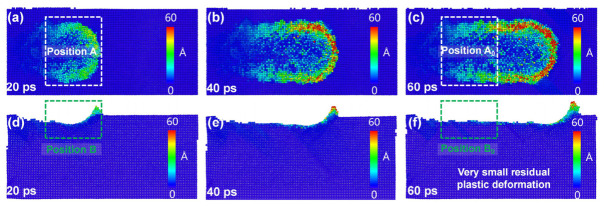
Atomic displacement contour maps of the scratched surface and subsurface at different times ((100) plane): (**a**) surface at 20 ps, (**b**) surface at 40 ps, (**c**) surface at 60 ps; (**d**) subsurface at 20 ps, (**e**) subsurface at 40 ps, (**f**) subsurface at 60 ps.

To further elucidate the deformation mechanisms and dislocation evolution of the FeCrNiCoCu high-entropy alloy during scratching, the atomic displacement evolution on both the scratched surface and subsurface was analyzed. As shown in Figure 6a,d, at a scratching time of 20 ps, significant atomic displacements are observed in both the surface and subsurface regions under the mechanical action of the abrasive, indicating pronounced plastic deformation. With increasing scratching time to 60 ps, as illustrated in Figure 6b,c,e,f, plastic deformation in both the surface and subsurface regions continues to develop. However, after the completion of scratching, only a small amount of residual plastic deformation remains in the subsurface region. A comparative analysis between positions A and A′, as well as B and B′, reveals that substantial elastic–plastic deformation occurs in both the surface and subsurface regions under the compressive and shear actions of the abrasive. As the abrasive progresses, the deformed regions exhibit a pronounced elastic recovery effect. Consequently, only a thin subsurface layer with minimal residual plastic deformation is retained after scratching. This behavior further confirms the excellent elastic recovery capability and deformation self-healing ability of the FeCrNiCoCu high-entropy alloy at the atomic scale.

#### 3.1.2. Effect of Crystallographic Orientation on the Machining Mechanism of FeCrNiCoCu High-Entropy Alloy

**Figure 7:**
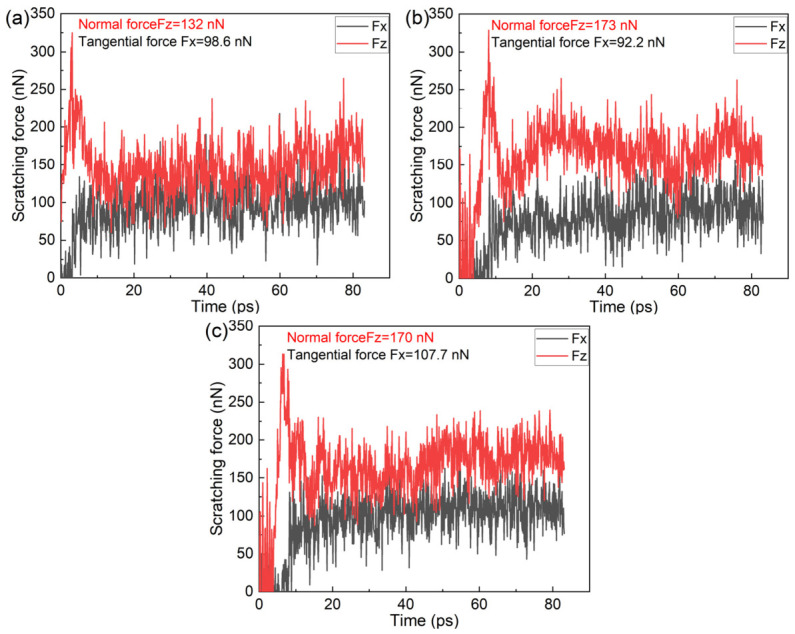
Friction force–time curves for different crystal planes: (**a**) (100) plane, (**b**) (110) plane, (**c**) (111) plane.

Crystalline materials generally exhibit pronounced anisotropy. For the face-centered cubic (fcc) FeCrNiCoCu high-entropy alloy, three primary crystallographic planes, namely (100), (110), and (111), are commonly observed. Differences in atomic arrangements among these planes can significantly influence their machining behavior. Scratching force is a key indicator for evaluating the stability of the scratching process and the material removal resistance. Therefore, the scratching force–time curves for the (100), (110), and (111) planes of the FeCrNiCoCu HEA were analyzed, as shown in Figure 7. Under a scratching velocity of 2 Å/ps, the force responses of different crystallographic planes are presented in Figure 7a–c. The results show that the scratching force mainly consists of two components: the normal force and the tangential force. Specifically, for the (100) plane, the normal and tangential forces are 132 nN and 98.6 nN, respectively. For the (110) plane, the corresponding values are 173 nN and 92.2 nN, while for the (111) plane, they are 170 nN and 107.7 nN, respectively. These results indicate that the (100) plane exhibits the lowest normal force and relatively low tangential force during scratching, suggesting that material removal on the (100) surface is easier compared to the (110) and (111) planes. This difference can be attributed to the distinct dislocation activities and lattice evolution behaviors within the deformation zone during scratching.

**Figure 8:**
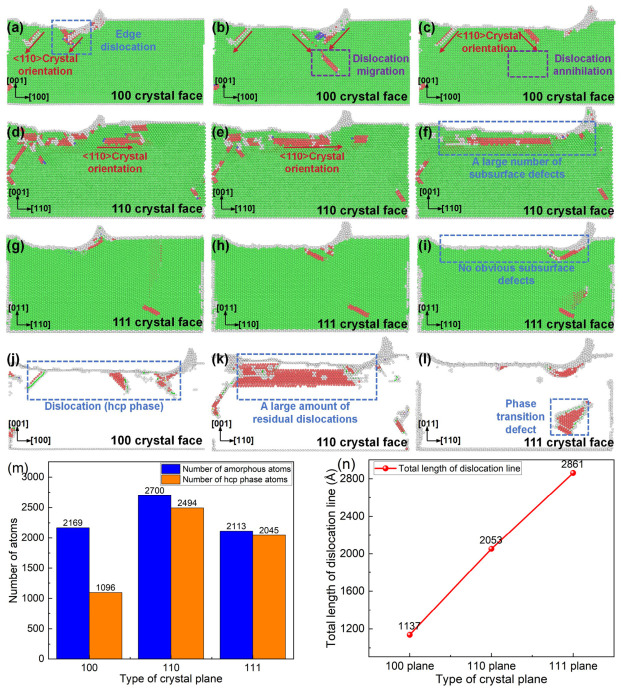
Subsurface lattice structures at different scratching times for various crystal planes: (**a**–**c**) (100) plane at 20 ps, 40 ps, and 60 ps; (**d**–**f**) (110) plane at 20 ps, 40 ps, and 60 ps; (**g**–**i**) (111) plane at 20 ps, 40 ps, and 60 ps; (**j**–**l**) phase transformation defects in the subsurface for all three planes; (**m**) variation in the number of amorphous and hcp atoms induced by scratching with crystal plane type; (**n**) variation in the total dislocation line length within the material after scratching with crystal plane type.

To investigate the effect of crystallographic orientation on the deformation mechanisms of the FeCrNiCoCu high-entropy alloy, CBN abrasive scratching simulations were performed on the three primary crystallographic planes, namely (100), (110), and (111). The scratching directions were aligned with the principal crystallographic directions of each plane, i.e., <100>, <110> and <111>, respectively. The lattice configurations and atomic displacement fields of both the scratched surface and subsurface for the three orientations are presented in Figure 8. As shown in Figure 8a–i, the dislocations generated during scratching on all three crystallographic planes are predominantly distributed along the <110> direction. This indicates that the dislocation nucleation mechanism in the FeCrNiCoCu HEA is primarily governed by its fcc lattice structure and is largely independent of crystallographic orientation. However, significant differences are observed in the subsurface dislocation evolution among the three planes.

For the (100) plane (Figure 8a–c), dislocations in the subsurface region migrate with the movement of the scratching-induced deformation zone, and pronounced dislocation annihilation occurs during the scratching process. As a result, only a small number of residual dislocations remain after scratching. In contrast, for the (110) plane (Figure 8d–f), a large number of dislocations persist throughout the entire subsurface region, with negligible annihilation observed as the deformation zone propagates. For the (111) plane (Figure 8g–i), although the shallow subsurface region shows minimal dislocation retention, distinct fcc-to-hcp phase transformation defects are observed in the deeper subsurface layers. The distribution of hcp atoms in the subsurface region (Figure 8j–l) further supports these observations. Only a small number of hcp phase defects are present beneath the (100) surface, whereas a substantial amount of phase transformation defects are observed in the (110) subsurface. For the (111) plane, phase transformation defects are mainly localized in the deeper subsurface region. Since dislocations in fcc materials are closely associated with hcp atomic structures, the number of hcp atoms can be used as an indicator of dislocation density. Quantitatively, as shown in Figure 8m, the number of hcp atoms generated during scratching is 1096 for the (100) plane, 2494 for the (110) plane, and 2045 for the (111) plane. Furthermore, the total dislocation line length within the same volume, which reflects the dislocation density, is presented in Figure 8n. After scratching, the total dislocation line lengths are 1137 Å for the (100) plane, 2053 Å for the (110) plane, and 2861 Å for the (111) plane. Overall, the MD simulation results indicate that, for the FeCrNiCoCu high-entropy alloy, the (100) plane exhibits superior lattice structural recovery, with only a small number of residual dislocations and phase transformation defects remaining after scratching. In contrast, the (110) plane shows relatively poor self-healing ability, leading to significant dislocation accumulation and phase transformation defects in the subsurface region.

**Figure 9:**
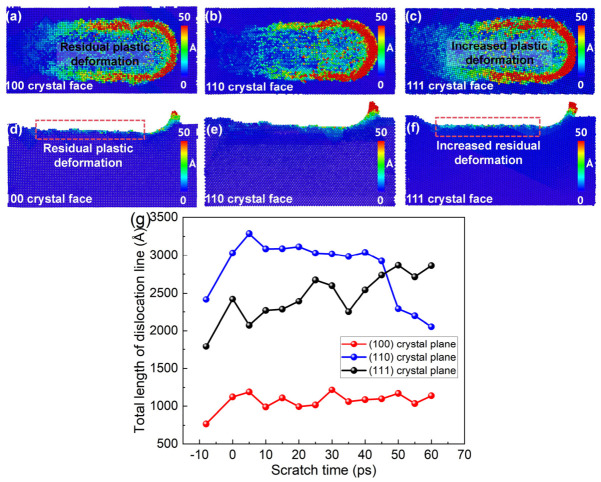
Atomic displacement contour maps for the scratched surface of different crystal planes: (**a**) (100) plane, (**b**) (110) plane, (**c**) (111) plane; atomic displacement contour maps for the scratched subsurface of different crystal planes: (**d**) (100) plane, (**e**) (110) plane, (**f**) (111) plane; (**g**) variation in the total dislocation line length in the scratched subsurface over scratching time for different crystal planes.

As shown in Figure 9, under identical scratching conditions, noticeable differences in atomic displacement and deformation behavior are observed among the three crystallographic planes. The (100) plane exhibits the smallest residual plastic deformation, whereas the (111) and (110) planes show significantly larger residual plastic deformation in the subsurface region. This indicates that the atomic arrangement of crystallographic planes is a key factor governing the machining-induced deformation behavior of the FeCrNiCoCu high-entropy alloy. Dislocations play a dominant role in the plastic deformation of metallic materials. A higher dislocation density within the deformation zone generally leads to more intense dislocation motion and, consequently, greater plastic deformation [35]. Figure 9g presents the evolution of dislocation density in the subsurface region during scratching for different crystallographic planes. The results show that the total dislocation line length beneath the (100) surface remains consistently lower than that of the (110) and (111) surfaces throughout the entire scratching process. This observation suggests that the differences in plastic deformation behavior among the three crystallographic planes are primarily attributed to variations in dislocation density within the scratching-induced deformation zone. Specifically, the higher dislocation densities in the subsurface regions of the (110) and (111) planes result in more pronounced plastic deformation compared to the (100) plane.

### 3.2. Polycrystalline FeCrNiCoCu High-Entropy Alloy

#### 3.2.1. Machining Mechanism of Polycrystalline FeCrNiCoCu High-Entropy Alloy

To investigate the deformation and material removal mechanisms of polycrystalline FeCrNiCoCu high-entropy alloy, the lattice evolution in the subsurface region during CBN abrasive scratching was analyzed using OVITO.

**Figure 10:**
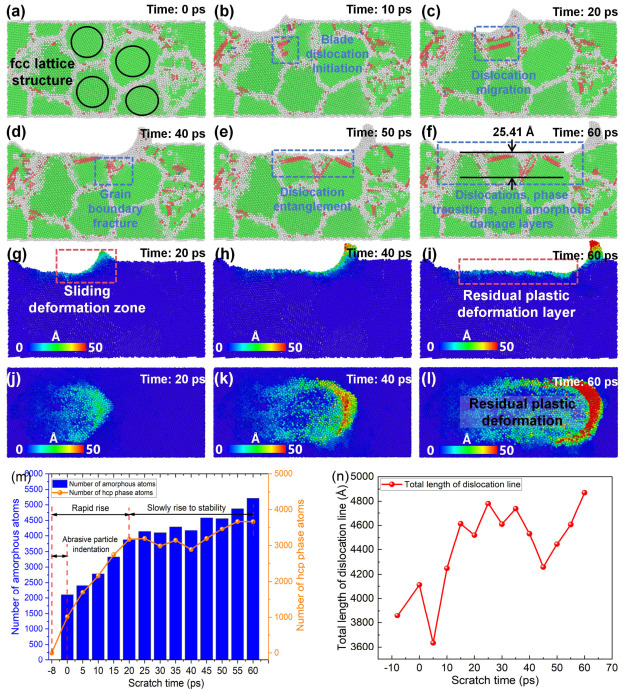
Subsurface lattice structures and the time evolution of scratching-induced amorphization and hcp atoms in the polycrystalline model (Grain size: 40 Å). Subsurface lattice structures at different scratching times: (**a**) before scratching, (**b**) 10 ps, (**c**) 20 ps, (**d**) 40 ps, (**e**) 50 ps, (**f**) 60 ps; (**g**,**h**) atomic displacement cloud maps of the scratching subsurface at different times; (**i**–**l**) atomic displacement cloud maps of the scratching surfaces at different times;(**m**) variation in the number of amorphous and hcp atoms induced by scratching over time; (**n**) variation in the total dislocation line length within the material after scratching over time.

As shown in Figure 10a, prior to scratching, the grains and grain boundaries in the alloy subsurface exhibit well-defined morphologies, with no observable internal defects. Upon indentation by the abrasive particle, dislocations begin to nucleate within the subsurface lattice, as shown in Figure 10b. With increasing scratching time up to 30 ps, the dislocations in the subsurface region migrate and multiply (Figure 10c). During this process, some dislocations are absorbed by grain boundaries, resulting in dislocation annihilation, while others penetrate and disrupt the grain boundaries (Figure 10d). Sustained plastic deformation in the subsurface during scratching is primarily governed by dislocation migration and grain boundary rupture. Figure 10e shows that dislocation entanglement occurs in the subsurface, which serves as the primary origin of machining-induced defects. After completion of scratching, a dislocation damage layer with a depth of 25.41 Å is observed beneath the surface (Figure 10f). Quantitative analysis of atomic structures indicates that the number of amorphous atoms increases from 2101 to 5220, while the number of hcp-phase atoms rises from 1011 to 3666 throughout the scratching process (Figure 10m). Compared to single-crystal FeCrNiCoCu, the polycrystalline alloy exhibits more severe amorphization and phase transformation in the subsurface. This is attributed to the random orientation of grains in the polycrystalline material, which facilitates nucleation of amorphous and phase transformation defects both within grains and at grain boundaries. Atomic displacement maps (Figure 10g–l) further reveal pronounced plastic deformation on both the scratched surface and subsurface. After scratching, a well-defined plastic deformation layer exists in the surface and subsurface regions, with a deformation magnitude notably larger than that observed in single-crystal FeCrNiCoCu. Correspondingly, the total dislocation line length in the subsurface increases from 3858 Å to 4870 Å during scratching (Figure 10n). A comparison with the single-crystal model shows that, at all stages of scratching, the total dislocation line length in the polycrystalline model is significantly higher. This indicates that dislocation density is the primary factor controlling the extent of plastic deformation, and the more intense dislocation motion in the polycrystalline model explains its larger residual plastic deformation.

#### 3.2.2. Effect of Grain Size on the Machining Mechanism of Polycrystalline FeCrNiCoCu High-Entropy Alloy

**Figure 11:**
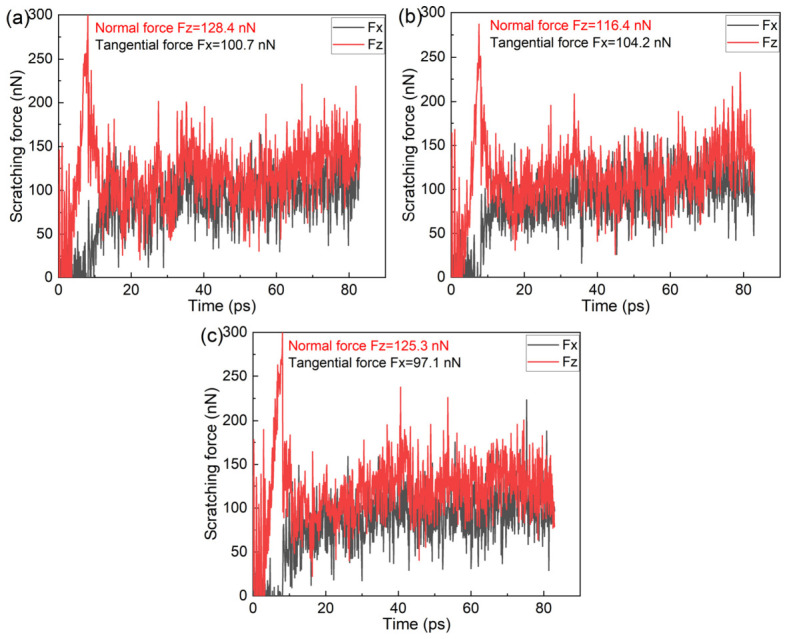
Friction force–time curves for different average grain sizes: (**a**) average grain size: 35 Å, (**b**) average grain size: 40 Å, (**c**) average grain size: 45 Å.

Grain size is generally recognized as a critical factor influencing the precision machining performance of polycrystalline materials. The scratching force–time responses of polycrystalline FeCrNiCoCu high-entropy alloys with different average grain sizes were compared to investigate this effect. As shown in Figure 11a–c, for the model with an average grain size of 35 Å, the normal force reaches 128.4 nN and the tangential force is 100.7 nN. For the 40 Å grain model, the normal and tangential forces are 116.4 nN and 104.2 nN, respectively, while for the 45 Å grain model, the corresponding forces are 125.3 nN and 97.1 nN. A comprehensive comparison indicates that the model with 35 Å grains exhibits the highest scratching force. This suggests that the smaller the average grain size in the polycrystalline FeCrNiCoCu alloy, the higher the machining force required during scratching. This behavior can be attributed to more intense dislocation activity within the deformation zone in smaller grains, which enhances resistance to material removal.

**Figure 12:**
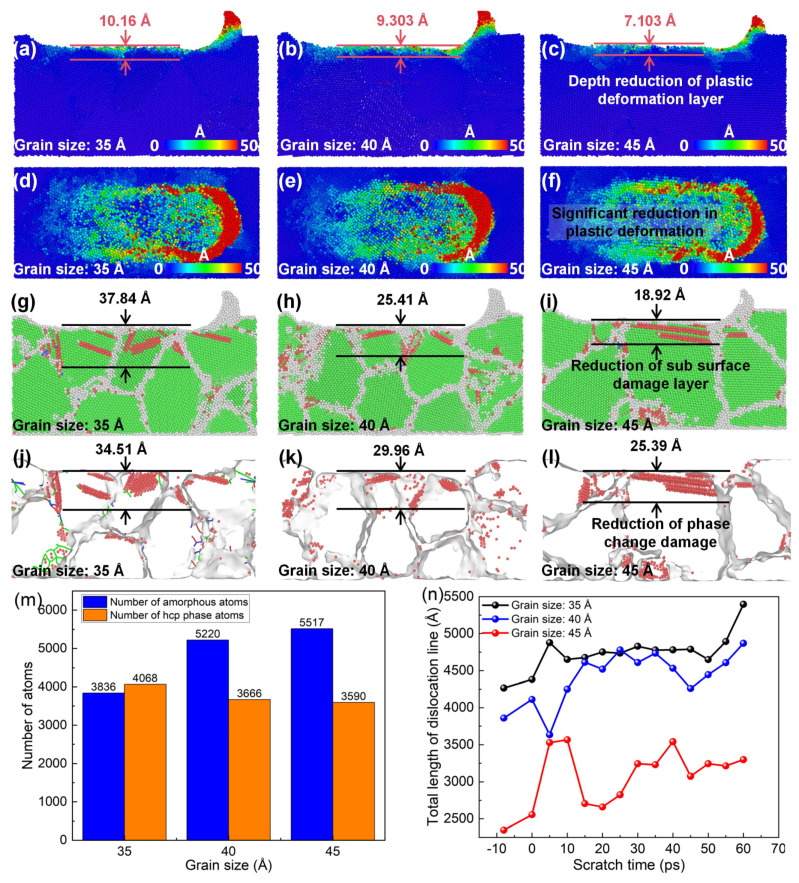
Atomic displacements and lattice structures in the scratched subsurface for different grain sizes. Atomic displacements on the scratched surface: (**a**) average grain size: 35 Å, (**b**) average grain size: 40 Å, (**c**) average grain size: 45 Å; atomic displacements in the scratched subsurface: (**d**) average grain size: 35 Å, (**e**) average grain size: 40 Å, (**f**) average grain size: 45 Å; (**g**–**l**) subsurface lattice structures and phase transformation defects for different grain sizes; (**m**) variation in scratching-induced amorphous and hcp atoms with grain size; (**n**) variation in total dislocation line length in the scratched subsurface over time for different grain sizes.

The influence of different average grain sizes on the subsurface lattice morphology, dislocation defects, and plastic deformation of polycrystalline FeCrNiCoCu high-entropy alloys during scratching was analyzed, and the results are shown in Figure 12. As illustrated by the atomic displacement maps in Figure 12a–f, increasing the average grain size leads to a reduction in the residual plastic deformation of both the scratched surface and the subsurface. Specifically, the depth of the residual plastic deformation layer in the subsurface decreases from 10.16 Å to 7.013 Å. Correspondingly, as shown in Figure 12k, the total dislocation line length in the subsurface at each stage of scratching decreases with increasing grain size. This indicates that appropriately increasing the grain size can effectively reduce the dislocation density in the scratching subsurface, minimize residual plastic deformation, and improve machining quality. A comparison of Figure 11 and Figure 12k suggests that the higher scratching forces observed in models with smaller grain sizes are primarily caused by the higher dislocation density within the deformation zone.

Furthermore, Figure 12g–l demonstrate that as the average grain size increases from 35 Å to 45 Å, both dislocation and phase transformation damage in the scratching subsurface are reduced. The depth of the subsurface lattice damage layer decreases from 37.84 Å to 18.92 Å, while the depth of the hcp phase transformation defect layer decreases from 34.51 Å to 25.39 Å. As shown in Figure 12j, under the same scratching parameters, the number of amorphized atoms in the subsurface increases from 3836 to 5517 with increasing grain size, whereas the number of hcp phase atoms decreases from 4068 to 3590. In crystalline materials, amorphization can be regarded as a form of material removal. The increase in amorphized atoms with larger grain size indicates that increasing the grain size can enhance the material removal rate of FeCrNiCoCu high-entropy alloys. Since dislocations and phase transformation defects primarily exist in the hcp phase, the reduction in hcp atoms with increasing grain size demonstrates that larger grains effectively mitigate subsurface phase transformation damage.

Overall, the MD simulations indicate that grain size is a critical factor controlling the machining-induced deformation of FeCrNiCoCu high-entropy alloys. Increasing the grain size reduces both subsurface phase transformation defects and plastic deformation, thereby improving the overall machining quality.

### 3.3. Comparison Between FeCrNiCoCu HEA and Conventional FCC Metal (Cu)

To further evaluate the validity of the present molecular dynamics framework and to quantitatively compare the machining behavior of FeCrNiCoCu high-entropy alloys with conventional metallic materials, additional nanoscratching simulations were performed using pure Cu single-crystal and polycrystalline models as reference systems. Except for the workpiece material, all simulation parameters and scratching conditions were identical to those used in the FeCrNiCoCu scratching models shown in Figure 3a,b. The pure Cu workpiece adopts a face-centered cubic (FCC) lattice structure with a lattice constant of 3.615 Å. The interactions between Cu atoms were described using the embedded atom method (EAM) potential, while the interactions between Cu atoms and B/N atoms in the CBN abrasive were modeled using the Lennard–Jones (L–J) potential with the same parameters listed in Table 4.

**Figure 13:**
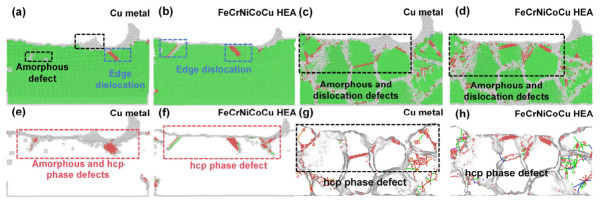
Comparison of subsurface lattice morphologies during scratching between FeCrNiCoCu high-entropy alloy and Cu metal: (**a**,**e**) scratched single-crystal Cu; (**b**,**f**) scratched single-crystal FeCrNiCoCu HEA; (**c**,**g**) scratched polycrystalline Cu; (**d**,**h**) scratched polycrystalline FeCrNiCoCu HEA. The upper images show subsurface lattice morphologies, while the lower images show hcp-phase defects.

Figure 13a,b show the subsurface lattice morphology of scratched single-crystal Cu and single-crystal FeCrNiCoCu HEA under identical scratching conditions. Compared with the HEA, the scratched subsurface of single-crystal Cu exhibits significantly more severe amorphization and dislocation defects. In addition, as shown in Figure 13e,f, the number of hcp-phase atoms beneath the scratched surface of Cu is higher than that observed in the FeCrNiCoCu HEA. Since HCP atoms in FCC materials are strongly associated with dislocation activity and phase transformation, these results indicate that the HEA possesses superior resistance to subsurface defect formation and lattice damage during scratching. Similarly, Figure 13c,d,g,h compare the subsurface deformation behavior of polycrystalline Cu and polycrystalline FeCrNiCoCu HEA under the same scratching conditions and grain size. The results show that the polycrystalline Cu model generates significantly larger numbers of dislocations, amorphous atoms, and HCP-phase defects than the polycrystalline FeCrNiCoCu HEA. These comparative MD simulation results demonstrate that, relative to conventional FCC metals, FeCrNiCoCu high-entropy alloys exhibit significantly reduced subsurface defect formation, lower dislocation density, and weaker phase transformation during nanoscratching. Consequently, the HEA exhibits improved subsurface structural stability and higher machining surface quality.

Furthermore, the deformation characteristics observed in the pure Cu scratching simulations, including extensive HCP defect formation and pronounced dislocation evolution, are consistent with previously reported nanoscratching studies of FCC metals using EAM potentials. This agreement further supports the validity and reliability of the present EAM-based computational framework for investigating nanoscale deformation mechanisms in FeCrNiCoCu high-entropy alloys.

## 4. Influence of Elemental Composition on the Machining Mechanism of FeCrNiCoCu High-Entropy Alloys

Elemental composition is a key factor influencing the machining performance of multi-principal-element high-entropy alloys. Previous studies have indicated that Cu, Cr, and Fe are critical elements affecting the precision machining quality of FeCrNiCoCu high-entropy alloys [36,37,38]. To reveal the atomic-scale influence of these three elements on the nanomachining mechanisms of FeCrNiCoCu alloys, molecular dynamics simulations were conducted to investigate how variations in the content of Cu, Cr, and Fe affect the subsurface lattice evolution and plastic deformation during scratching.

### 4.1. Single-Crystal FeCrNiCoCu High-Entropy Alloy

**Figure 14:**
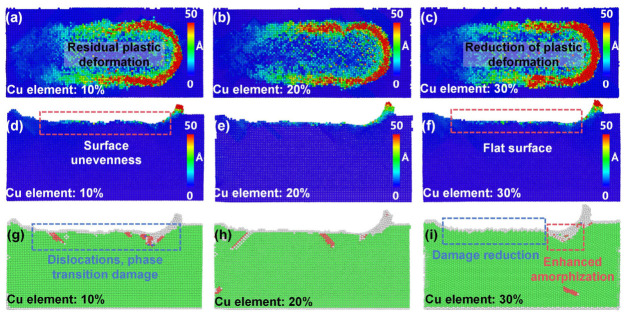

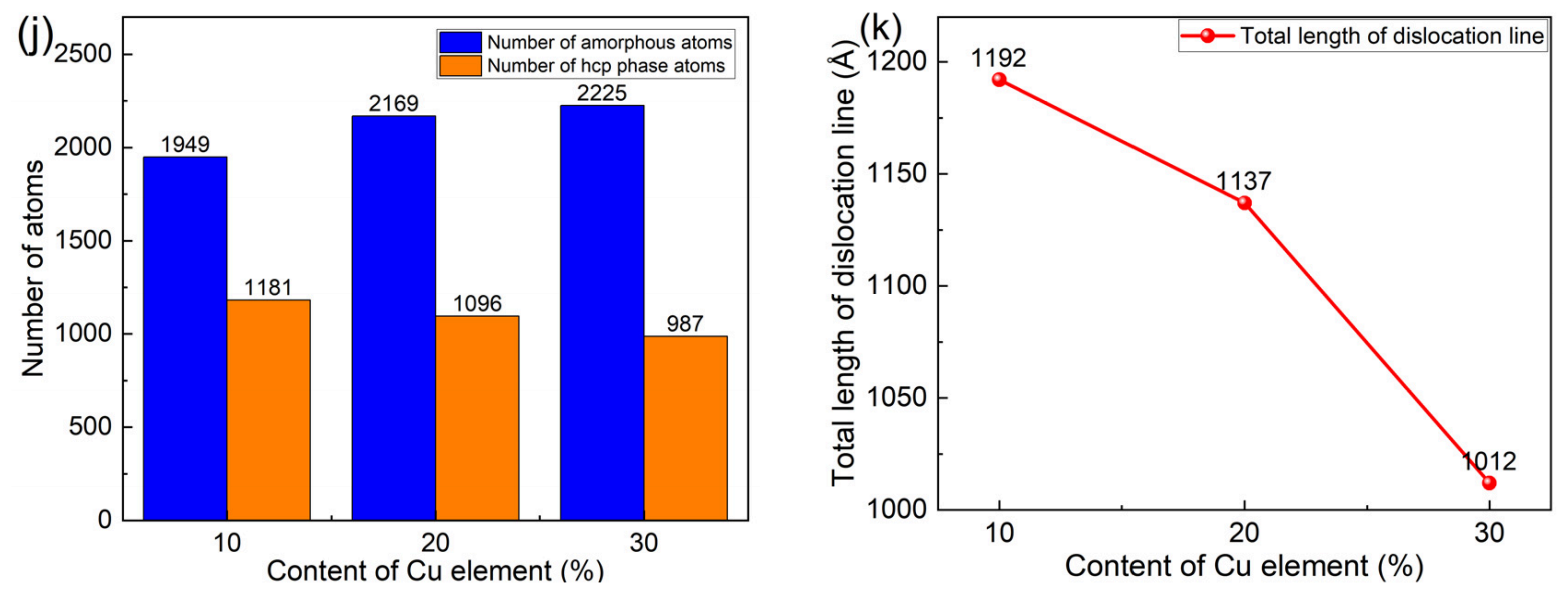
Atomic displacements, lattice structures, and phase-transformed atoms in the scratched subsurface of single-crystal FeCrNiCoCu alloys with different Cu contents (Grain size: 40 Å). (**a**–**f**) Variation in atomic displacements on the scratched surface and subsurface with Cu content; (**g**–**i**) Variation in lattice structures and phase transformation defects on the scratched surface and subsurface with Cu content; (**j**) Variation in scratching-induced amorphous and hcp atoms with Cu content; (**k**) Variation in total dislocation line length in the scratched subsurface with Cu content.

Figure 14 shows the subsurface lattice morphology and atomic displacement of single-crystal FeCrNiCoCu high-entropy alloys with varying Cu content after CBN particle scratching. As shown in Figure 14a–f, with an increase in Cu content from 10 at.% to 30 at.%, the surface plastic deformation of the alloy decreases, while the residual plastic deformation in the subsurface remains largely unchanged. However, the surface smoothness improves. Figure 14k further indicates that, as the Cu content increases, the total dislocation line length in the scratched subsurface decreases from 1190 Å to 1021 Å. This suggests that increasing the Cu content suppresses dislocation motion during the machining process, thereby reducing plastic deformation and improving the machined surface quality. Figure 14g–i show that with Cu content increasing from 10 at.% to 30 at.%, the number of edge dislocations in the shallow subsurface decreases after scratching. Notably, when Cu content reaches 30 at.%, dislocation defects appear in the deeper subsurface layers. Figure 14j reveals that, under consistent conditions, increasing Cu content results in an increase in the number of amorphous atoms from 1949 to 2225, while the number of hcp-phase atoms decreases from 1181 to 987. The increase in amorphous atoms suggests an enhanced material removal rate, whereas the decrease in hcp-phase atoms reflects a reduction in subsurface dislocations and phase transformation defects. These results indicate that a higher Cu content reduces subsurface dislocation and phase transformation damage, improving machining quality, while simultaneously enhancing material removal. The mechanism is that an increase in copper content suppresses the formation of dislocations in the deformation zone, thereby reducing subsurface plastic deformation and lattice defects, and improving processing quality.

**Figure 15:**
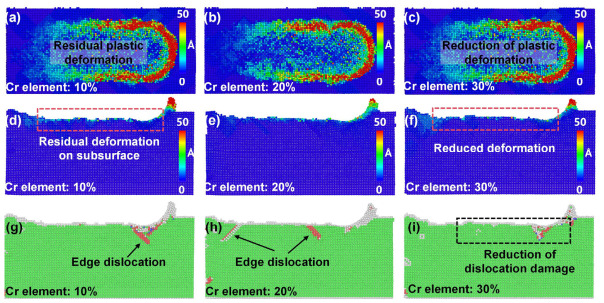

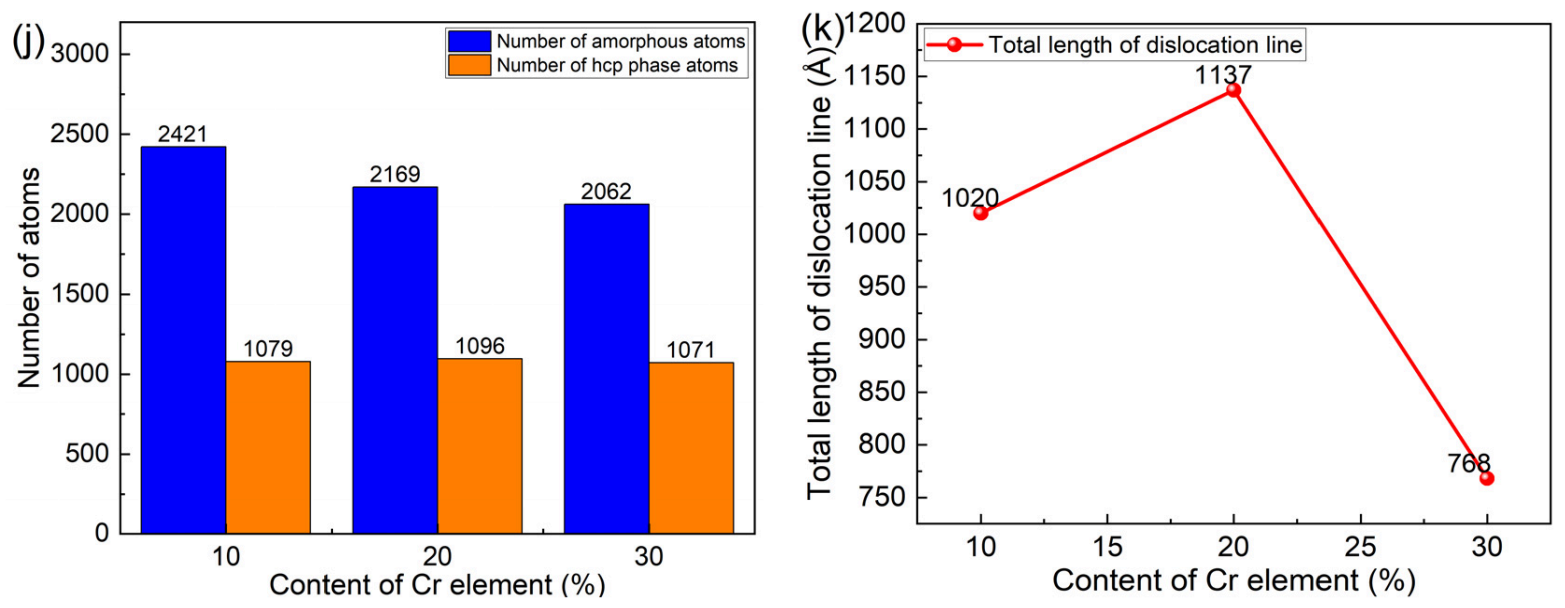
Atomic displacements, lattice structures, and phase-transformed atoms in the scratched subsurface of single-crystal FeCrNiCoCu alloys with different Cr contents (Grain size: 40 Å). (**a**–**f**) Variation in atomic displacements on the scratched surface and subsurface with Cr content; (**g**–**i**) Variation in lattice structures and phase transformation defects on the scratched surface and subsurface with Cr content; (**j**) Variation in scratching-induced amorphous and hcp atoms with Cr content; (**k**) Variation in total dislocation line length in the scratched subsurface with Cr content.

Figure 15 shows the lattice morphology and atomic displacement of the surface and subsurface of single-crystal FeCrNiCoCu high-entropy alloys with varying Cr content after CBN particle scratching. As observed from the atomic displacement maps in Figure 15a–f, under identical scratching conditions, increasing the Cr content from 10 at.% to 30 at.% significantly reduces the residual plastic deformation on both the scratched surface and the subsurface. Figure 15k shows that, with increasing Cr content, the total dislocation line length in the scratched subsurface decreases from 1026 Å to 768 Å. This indicates that higher Cr content suppresses dislocation motion in the scratching deformation zone, thereby reducing residual plastic deformation. Figure 15g–i further reveal that, with increasing Cr content, both amorphization and dislocation defects in the subsurface are markedly reduced, improving the lattice integrity. Figure 15j shows that, as Cr content increases from 10 at.% to 30 at.%, the number of amorphous atoms in the scratched subsurface decreases from 2421 to 2062, while the number of hcp-phase atoms decreases slightly from 1079 to 1071. These MD simulation results indicate that an appropriate increase in Cr content effectively mitigates subsurface dislocations, phase transformation defects, and plastic deformation in FeCrNiCoCu high-entropy alloys, thereby enhancing the machining quality.

**Figure 16:**
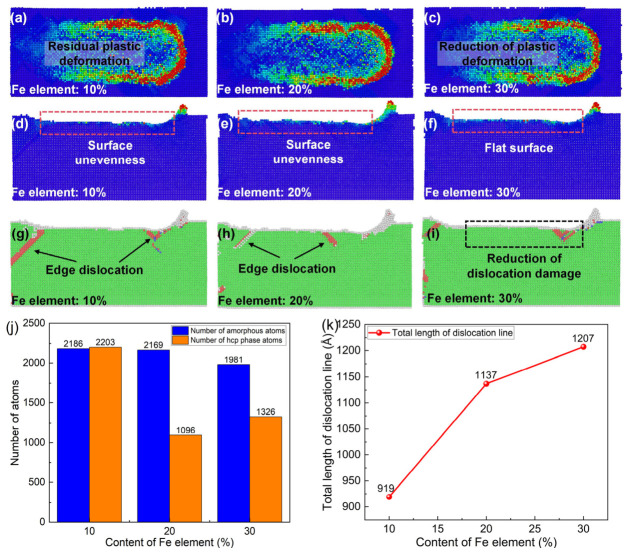
Atomic displacements, lattice structures, and phase-transformed atoms in the scratched subsurface of single-crystal FeCrNiCoCu alloys with different Fe contents (Grain size: 40 Å). (**a**–**f**) Variation in atomic displacements on the scratched surface and subsurface with Fe content; (**g**–**i**) Variation in lattice structures and phase transformation defects on the scratched surface and subsurface with Fe content; (**j**) Variation of scratching-induced amorphous and hcp atoms with Fe content; (**k**) Variation in total dislocation line length in the scratched subsurface with Fe content.

Figure 15 shows the atomic displacement and lattice morphology of the surface and subsurface of single-crystal FeCrNiCoCu high-entropy alloys with varying Fe content after CBN particle scratching. As observed in Figure 16a–f, under identical scratching conditions, increasing the Fe content from 10 at.% to 30 at.% does not significantly alter the atomic displacement on the scratched surface, but the residual plastic deformation in the subsurface increases. Figure 16k shows that, with increasing Fe content, the total dislocation line length in the scratched subsurface rises from 919 Å to 1207 Å, indicating that excessive Fe content intensifies dislocation motion in the subsurface, leading to larger residual plastic deformation. Figure 16g–i further reveal that, despite the increased dislocation motion, the number of dislocation and phase transformation defects in the scratched subsurface decreases with increasing Fe content. When Fe content is 10 at.%, deep dislocations are evident in the subsurface, whereas at 30 at.% Fe, the depth of the dislocation defect layer is significantly reduced. This indicates that an appropriate increase in Fe content can effectively suppress dislocation generation and motion, reducing subsurface defects. Figure 16j shows that, as Fe content increases from 10 at.% to 30 at.%, the number of amorphous atoms in the scratched subsurface decreases from 2186 to 1981, while the number of hcp-phase atoms decreases from 2203 to 1326. The simultaneous reduction in both amorphous and hcp-phase atoms indicates a concurrent decrease in subsurface amorphization and phase transformation damage. Overall, the MD simulation results indicate that an appropriate increase in Fe content can reduce lattice phase transformation damage in the scratched subsurface of single-crystal FeCrNiCoCu high-entropy alloys, thereby improving machining quality. Although higher Fe content slightly enhances dislocation motion and increases plastic deformation in the subsurface, it also improves the lattice defect annihilation and structural recovery, ultimately reducing subsurface lattice defects.

Overall, MD simulations reveal that Cu, Cr, and Fe contents significantly affect the machining behavior of single-crystal FeCrNiCoCu alloys. Higher Cu content primarily improves surface quality and material removal by suppressing subsurface dislocation motion, higher Cr content enhances lattice integrity and reduces subsurface defects, while moderate increases in Fe content enhance lattice self-healing and reduce phase transformation damage. These results highlight the critical role of elemental composition in controlling dislocation behavior, phase transformation, and plastic deformation during precision machining of FeCrNiCoCu high-entropy alloys.

### 4.2. Polycrystalline FeCrNiCoCu High-Entropy Alloy

In industrial applications, high-entropy alloys are often used in polycrystalline form. Understanding the influence of elemental composition on subsurface defect evolution during scratching is therefore critical for guiding precision machining of polycrystalline FeCrNiCoCu alloys.

**Figure 17:**
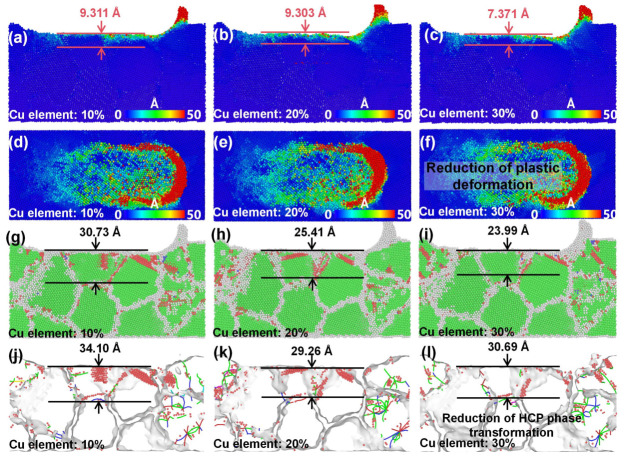

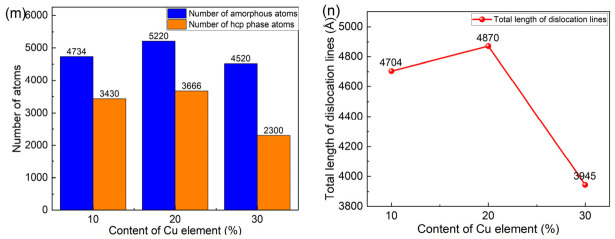
Atomic displacements, lattice structures, and phase-transformed atoms in the scratched subsurface of polycrystalline FeCrNiCoCu alloys with different Cu contents. (**a**–**f**) Variation in atomic displacements on the scratched surface and subsurface with Cu content; (**g**–**i**) Variation in subsurface lattice structures and phase transformation defects with Cu content; (**j**–**l**) Morphology of hcp phase transformations in the scratched subsurface with Cu content; (**m**) Variation in scratching-induced amorphous and hcp atoms with Cu content; (**n**) Variation in total dislocation line length in the scratched subsurface with Cu content.

Figure 17 shows the atomic displacement and lattice morphology of polycrystalline FeCrNiCoCu alloys with varying Cu content after CBN particle scratching. The magnitude of atomic displacement is a key indicator of plastic deformation. From the atomic displacement maps in Figure 17a–f, under identical scratching conditions, increasing the Cu content from 10 at.% to 30 at.% gradually reduces atomic displacements on both the scratched surface and subsurface. Correspondingly, the depth of residual subsurface plastic deformation layer decreases from 9.311 Å to 7.371 Å. This indicates that increasing Cu content effectively suppresses plastic deformation, reducing residual deformation on the scratched surface and improving machining quality. As shown in Figure 17n, the total dislocation line length in the subsurface decreases from 4704 Å to 3945 Å with increasing Cu content. The reduction in dislocation line length reflects a lower dislocation density, demonstrating that the suppression of plastic deformation by Cu is primarily achieved through limiting dislocation motion.

Figure 17g–l show the lattice and phase transformation defect evolution in the scratched subsurface for different Cu contents. Increasing Cu from 10 at.% to 30 at.% leads to a progressive reduction in amorphization, dislocation, and hcp-phase defects. Specifically, the depth of the amorphous layer decreases from 30.73 Å to 23.99 Å, and the depth of the hcp-phase defect layer decreases from 34.10 Å to 30.69 Å. In addition, Figure 17m shows that the number of amorphous atoms decreases from 4734 to 4520, and the number of hcp-phase atoms decreases from 3430 to 2300 as Cu content increases. These MD simulation results indicate that appropriately increasing Cu content can effectively reduce subsurface amorphization, dislocation, and phase transformation damage in polycrystalline FeCrNiCoCu alloys, leading to a thinner damaged layer and improved machining quality.

**Figure 18:**
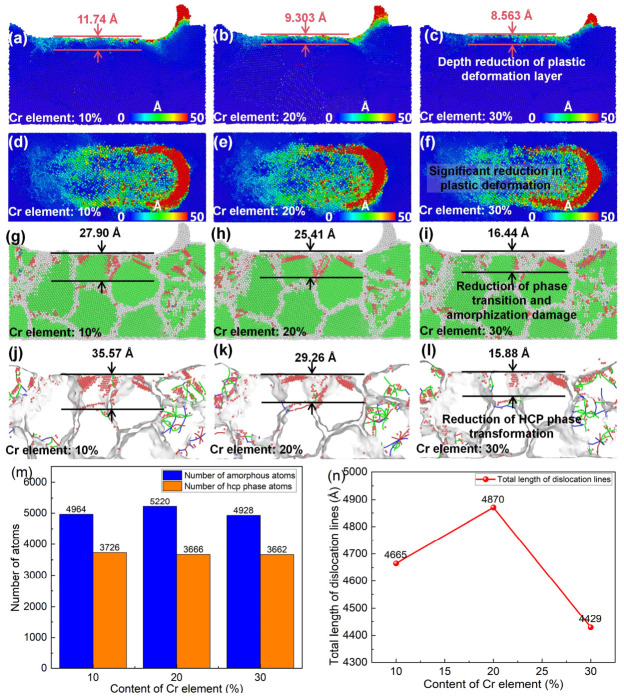
Atomic displacements, lattice structures, and phase-transformed atoms in the scratched subsurface of polycrystalline FeCrNiCoCu alloys with different Cr contents. (**a**–**f**) Variation in atomic displacements on the scratched surface and subsurface with Cr content; (**g**–**i**) Variation in subsurface lattice structures and phase transformation defects with Cr content; (**j**–**l**) Morphology of hcp phase transformations in the scratched subsurface with Cr content; (**m**) Variation in scratching-induced amorphous and hcp atoms with Cr content; (**n**) Variation in total dislocation line length in the scratched subsurface with Cr content.

Figure 18 shows the atomic displacement and lattice morphology of polycrystalline FeCrNiCoCu alloys with varying Cr content after CBN particle scratching. From the atomic displacement maps in Figure 18a–f, under identical scratching conditions, increasing Cr content from 10 at.% to 30 at.% gradually reduces residual plastic deformation on both the scratched surface and subsurface. The depth of the residual subsurface plastic deformation layer decreases from 11.74 Å to 8.563 Å, indicating that an appropriate increase in Cr content can suppress plastic deformation during scratching and improve machining quality. As shown in Figure 18n, the total dislocation line length in the subsurface decreases from 4665 Å to 4429 Å with increasing Cr content, demonstrating that Cr can also effectively reduce dislocation density in the scratched subsurface and, consequently, the residual plastic deformation. Figure 18g–l present the lattice and phase transformation defect evolution in the scratched subsurface for different Cr contents. Increasing Cr content from 10 at.% to 30 at.% leads to a progressive reduction in dislocation and hcp-phase defects, with the depth of the dislocation/amorphous damage layer decreasing from 27.90 Å to 16.44 Å and the depth of the hcp-phase defect layer decreasing from 35.57 Å to 15.88 Å. Figure 18m shows that the number of amorphous atoms remains nearly unchanged, while the number of hcp-phase atoms decreases from 3726 to 3662 as Cr content increases. These MD simulation results indicate that appropriately increasing Cr content can reduce dislocation and phase transformation damage in polycrystalline FeCrNiCoCu alloys, minimizing subsurface lattice defects and enhancing machining quality.

**Figure 19:**
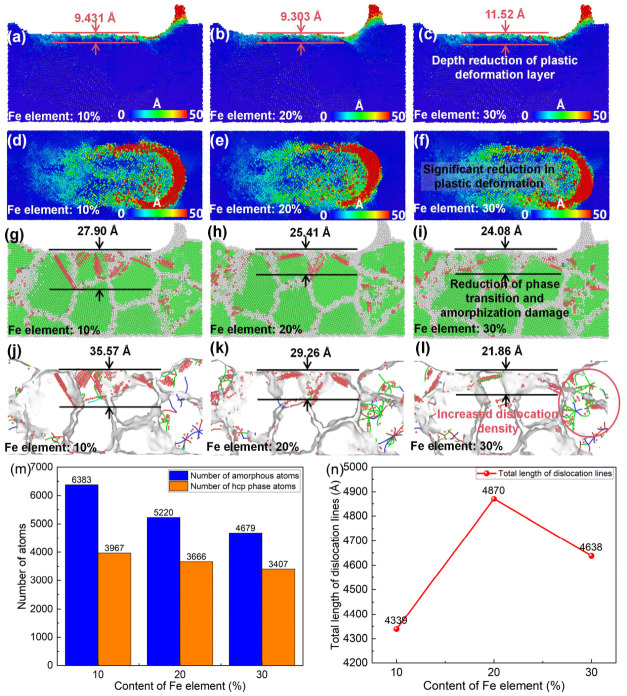
Atomic displacements, lattice structures, and phase-transformed atoms in the scratched subsurface of polycrystalline FeCrNiCoCu alloys with different Fe contents. (**a**–**f**) Variation in atomic displacements on the scratched surface and subsurface with Fe content; (**g**–**i**) Variation in subsurface lattice structures and phase transformation defects with Fe content; (**j**–**l**) Morphology of hcp phase transformations in the scratched subsurface with Fe content; (**m**) Variation in scratching-induced amorphous and hcp atoms with Fe content; (**n**) Variation in total dislocation line length in the scratched subsurface with Fe content.

Figure 19 shows the atomic displacement and lattice morphology of polycrystalline FeCrNiCoCu alloys with different Fe contents after CBN particle scratching. From Figure 19a–f, under identical scratching conditions, increasing Fe content from 10 at.% to 30 at.% does not significantly change the atomic displacement on the scratched surface, but the depth of the residual plastic deformation layer in the subsurface increases from 9.431 Å to 11.52 Å. This indicates that higher Fe content leads to increased residual plastic deformation in the scratched subsurface. As shown in Figure 19n, the total dislocation line length in the subsurface increases from 4339 Å to 4638 Å with increasing Fe content, suggesting that elevated Fe content raises the dislocation density in the scratched subsurface, which in turn amplifies plastic deformation. Figure 19g–l illustrate the evolution of amorphous and phase-transformed defects in the scratched subsurface. Increasing Fe content from 10 at.% to 30 at.% significantly reduces both amorphous and hcp-phase defects in the subsurface, including within the grain interiors and at grain boundaries. The depths of the damage layer and hcp-phase layer decrease substantially. Figure 19m shows that the number of amorphous atoms decreases from 6383 to 4679, while the number of hcp-phase atoms decreases from 3967 to 3407. These MD simulation results indicate that appropriately increasing Fe content can effectively reduce lattice phase transformation defects and residual dislocations in the subsurface of polycrystalline FeCrNiCoCu alloys, minimize amorphous and phase-transformed defects inside grains and at grain boundaries, and thus improve machining quality.

Overall, the MD simulations demonstrate that elemental composition plays a crucial role in controlling the machining response of polycrystalline FeCrNiCoCu high-entropy alloys. Higher Cu and Cr contents primarily act to suppress dislocation motion and plastic deformation, reducing subsurface defects and improving surface quality. In contrast, increasing Fe content slightly amplifies plastic deformation but substantially mitigates phase transformation and amorphization in the subsurface. These quantitative insights provide guidance for optimizing the elemental composition of polycrystalline FeCrNiCoCu alloys to balance plasticity, defect suppression, and machining quality in precision manufacturing. In addition, although the simulations were conducted under deterministic conditions, the observed trends are consistent with established deformation mechanisms.

Previous MD studies have shown that nanoscratching of FCC-structured HEAs typically produces significant HCP-phase defects and dense subsurface dislocation structures beneath the scratched surface. Moreover, the reported subsurface damage depth generally falls within the range of approximately 10–30 Å, while the scratching force is commonly distributed in the range of about 50–200 nN under comparable MD loading conditions and scratching scales [3,20,39]. Combined with the results shown in Figure 11, Figure 16, Figure 17 and Figure 18, the present simulations exhibit similar subsurface defect characteristics, plastic deformation behavior, and comparable ranges of scratching force and damage-layer depth. This consistency indicates that the current computational framework, including the selected interatomic potentials and loading conditions, provides physically reasonable descriptions of the nanoscale deformation behavior of FeCrNiCoCu HEAs.

Compared with previous MD studies focusing on isolated factors such as scratching parameters or general deformation behavior, the present work systematically reveals the coupled effects of crystallographic orientation, grain size, and elemental composition on nanoscale defect evolution and machinability in FeCrNiCoCu HEAs. The results show that machining performance is governed by the synergistic interaction between dislocation activity, phase transformation, and lattice recovery behavior. Specifically, the (100) orientation, relatively larger grain sizes, and increased Cu/Cr contents effectively suppress subsurface defect accumulation and phase transformation damage, while moderate Fe enrichment promotes lattice self-healing. These findings establish direct structure–machinability relationships and provide atomistic guidance for optimizing the microstructure and composition of machinable HEAs.

Although the simulations successfully capture the coupled effects of crystallographic orientation, grain size, and elemental composition on the scratching behavior of FeCrNiCoCu high-entropy alloys, the current model still represents an idealized random solid solution combined with simplified LJ-based abrasive–substrate interactions. In real HEAs, local chemical heterogeneity, such as short-range order and elemental segregation, may significantly influence dislocation motion, stacking-fault formation, and energy dissipation, while the simplified interfacial description may also affect the quantitative values of friction force and subsurface damage.

More broadly, the nanoscale simulation domain, extremely high scratching velocity, finite boundary conditions, and simplified interaction potentials collectively introduce deviations from realistic machining environments. Therefore, the quantitative outputs (e.g., friction force, damage depth, defect density, and temperature rise) should not be directly interpreted as exact predictions for macroscopic processes. Nevertheless, since all simulations are performed within a consistent computational framework and the dominant response is governed by the intrinsic lattice behavior of the HEA substrate, the comparative trends remain qualitatively reliable. Accordingly, this work should be viewed as an atomistic mechanistic study that provides insight into nanoscale deformation and defect evolution in FeCrNiCoCu HEAs, complementing rather than replacing experimental and continuum-scale investigations. Future work will incorporate chemically heterogeneous structures and more advanced interatomic potentials to better capture realistic nanomachining behavior.

It should be noted that the present FeCrNiCoCu high-entropy alloy models were constructed as idealized random solid solutions without considering local chemical ordering or short-range order (SRO) effects. Previous studies have shown that related alloy systems, such as NiCoCr and FeCrNiCo alloys, may exhibit semi-ordered atomic structures or SRO behavior at relatively low temperatures [40,41,42,43], which can significantly influence stacking-fault energy, dislocation mobility, lattice distortion, and deformation behavior. Recent investigations further suggested that SRO may enhance dislocation pinning and increase the roughness of dislocation slip pathways, thereby improving deformation resistance and suppressing localized plasticity. Therefore, if SRO-containing FeCrNiCoCu models were employed in the present nanoscratching simulations, stronger resistance to dislocation nucleation and propagation, reduced subsurface defect density, and lower residual plastic deformation would likely be expected compared with the fully random solid-solution models adopted in this work. Although such effects are beyond the scope of the present study, incorporating chemically ordered or SQS-based HEA models into nanoscale machining simulations would be an important direction for future research.

## 5. Conclusions

The main conclusions on how crystallography, grain size, and elemental composition affect FeCrNiCoCu HEA machining are summarized below:

(1) Single-crystal FeCrNiCoCu HEAs: Dislocations nucleate along <110> directions and largely annihilate after scratching. The (100) plane shows minimal residual dislocations and phase transformation, demonstrating superior elastic recovery and lattice self-healing.
(2) Crystallographic orientation effects: Plane-specific atomic arrangements lead to distinct dislocation density, phase transformation, and residual deformation. The (100) plane provides the most favorable machining performance.
(3) Grain size effects: Smaller grains increase dislocation density and machining forces, whereas larger grains enhance elastic recovery and reduce subsurface damage.
(4) Elemental composition effects: Cu: Higher content suppresses dislocation motion, improves material removal, and enhances surface quality in both single- and polycrystals. Cr: Higher content strengthens lattice integrity, reduces subsurface defects, and suppresses dislocation-induced plasticity. Fe: Increasing Fe slightly amplifies plastic deformation but significantly reduces phase transformation and amorphization, aiding lattice self-healing.
(5) Overall insights: Crystallographic orientation, grain size, and elemental composition collectively control dislocation evolution, phase transformation, and plastic deformation during machining. The present findings provide atomistic insights into the deformation and defect evolution behavior of FeCrNiCoCu high-entropy alloys during nanoscratching and establish qualitative structure–machinability relationships that may assist future experimental and multiscale investigations on HEA precision machining.
(6) This work extends conventional MD nano-scratching studies by establishing a unified structure–deformation–machinability relationship for FeCrNiCoCu high-entropy alloys through the combined analysis of crystallographic orientation, grain size, and elemental composition. The results indicate that dominant (100)-oriented grains, relatively coarse grain structures, and Cu/Cr-rich compositions can effectively reduce subsurface damage and improve surface integrity during precision machining. These findings provide both atomistic insights into nanoscale deformation mechanisms and practical guidance for the microstructural design of machinable HEAs.

## Figures and Tables

**Table 1:** Elemental compositions for different Fe contents (at.%).

Type	Fe	Cr	Ni	Co	Cu
Reduce Fe content	10	22.5	22.5	22.5	22.5
Increase Fe content	30	17.5	17.5	17.5	17.5

**Table 2:** Settings of simulation parameters.

Material Properties	Crystal Type	Size	Number of Atoms	Lattice Structure
CBN grinding particle	Single crystal	Radius: 25 Å	B atoms: 5594 N atoms: 5576	cubic
FeCrNiCoCu high entropy alloy	Single crystal	200 Å × 90 Å × 100 Å	Fe atoms: 27,750, Ni atoms: 27,750, Cr atoms: 27,750, Co atoms: 27,750, Cu atoms: 27,750	fcc
	Polycrystalline			
Potential function	Fe, Ni, Cr, Co, Cu: EAM; B-B, B-N, N-N: ExTeP; Fe-B, Ni-B, Cr-B, Co-B, Cu-B, Fe-N, Ni-N, Cr-N, Co-N, Cu-N: L-J.			
Time step	1 fs			
Temperature	297 K			
Penetration depth	7.5 Å			
Friction speed	Multi-speed, velocity: 200 m/s			

**Table 3:** Parameters of ExTeP potential [30].

Parameters	Eb (eV/c.u.)	B2D (N/m)	C11,2D (N/m)	μ2D (N/m)	E2D (N/m)	ν2D (N/m)
Value	−13.38	164	277	113	267	0.186

**Table 4:** Parameters of Lennard–Jones potential (L-J) [32,33].

Interaction Pairs	σ (Å)	ε (eV)	Interaction Pairs	σ (Å)	ε (eV)
N-Fe	2.9293	0.06460	B-Fe	3.0546	0.04656
N-Cr	2.9390	0.06309	B-Cr	3.064	0.04548
N-Ni	2.8520	0.01713	B-Ni	2.974	0.01235
N-Co	3.1000	0.03449	B-Co	3.233	0.00673
N-Cu	2.9400	0.05697	B-Cu	3.066	0.04106

## Data Availability

The original contributions presented in this study are included in the article. Further inquiries can be directed to the corresponding author.
